# Common animal models lack a distinct glenoid labrum: a comparative anatomy study

**DOI:** 10.1186/s40634-021-00383-6

**Published:** 2021-08-16

**Authors:** Christopher J. Como, Benjamin B. Rothrauff, Peter G. Alexander, Albert Lin, Volker Musahl

**Affiliations:** Department of Orthopaedic Surgery, University of Pittsburgh, 3350 Terrace Street, Pittsburgh, PA 15213 USA

**Keywords:** Shoulder, Glenoid labrum, Animal model, Tissue engineering

## Abstract

**Purpose:**

Development and validation of an animal model of labral healing would facilitate translation of novel surgical and biological strategies to improve glenolabral healing. The purpose of this study was to characterize the anatomic and histological properties of the shoulder labrum in rat, rabbit, dog, pig, goat, and humans. Given the demonstrated similarities in size and structural morphology in other joints, it was hypothesized that the goat glenoid with surrounding capsulolabral complex would most closely resemble that of humans in terms of dimensions and structure, as observed grossly and histologically.

**Methods:**

Cadaveric glenohumeral joints from rats (*n* = 8), New Zealand white rabbits (*n* = 13), Mongrel dogs (*n* = 9), Spanish goats (*n* = 10), Yorkshire pigs (n = 10), and humans (n = 9) were freshly harvested. Photographs were taken of the glenoid with its surrounding capsulolabral complex. Linear dimensions of the glenoid articular surface were measured. It was determined where the capsulolabral complex was continuous with, or recessed from, the articular glenoid surface. The glenoid was divided into 6 equal segments radiating out toward 12, 2, 4, 6, 8, and 10 o’clock positions. Samples were sectioned and stained with Safranin O/Fast green and Mallory Trichrome. Insertion of the capsulolabral tissue onto the glenoid was qualitatively assessed and compared with gross morphology.

**Results:**

Dimensions of the goat glenoid most closely paralleled dimensions of the human glenoid. A capsulolabral complex was continuous with the glenoid surface from ~ 9 to 12 o’clock in the rats, 7 to 12 o’clock in rabbits, 5 to 12 o’clock in the dogs, and 9 to 12 o’clock in goats, 6 to 12 o’clock in pigs, and 2 to 8 o’clock in humans. In contrast to humans, no other species demonstrated an organized fibrocartilaginous labrum either macroscopically or histologically.

**Conclusion:**

The animals in the present study did not possess a discrete fibrocartilaginous labrum by gross or histological evaluation, as directly compared to humans. While models using these animals may be acceptable for examining other shoulder pathologies, they are not adequate to evaluate labral pathology.

**Level of evidence:**

Basic Science Study; Anatomy and Histology; Cadaveric Animal Model.

**Supplementary Information:**

The online version contains supplementary material available at 10.1186/s40634-021-00383-6.

## Introduction

Given the inherent instability of the glenohumeral joint, traumatic shoulder dislocation is particularly common and can cause damage to the soft tissues surrounding the joint [13, 24]. The glenoid labrum is damaged in more than 70% of traumatic shoulder dislocations and often fails to heal without intervention [4, 7]. Similarly, the joint capsule is routinely stretched during instability events, with residual laxity permitting excessive humeral translation and recurrent subluxation [16]. Recurrent dislocation is most commonly treated with surgical repair to prevent continued instability and subsequent joint degeneration [2, 6, 12, 18, 22, 29]. However, high rates of recurrent instability have been reported after labral repair [20, 23, 30]. Repair failure may in part be due to insufficient labral healing, either within the labral body or at the glenoid-labrum interface. Tissue engineering strategies have shown promise in preclinical studies in restoring structure and function following injury of the meniscus and rotator cuff [32, 33], but similar strategies have been seldom applied to capsulolabral injuries [25, 27, 36].

The development of improved surgical techniques and tissue engineering strategies for enhanced capsulolabral repair would be greatly facilitated by the use of animal models. Unfortunately, most model animals are quadrupeds that use their forelimbs for weight-bearing during locomotion and have limited overhead activity, differing greatly from humans. Additionally, some quadrupeds rarely use their upper limbs for functional tasks (i.e. goat and pig), while others use their arms to gather and eat food (i.e. rat and rabbit) [22]. Despite these functional variations within quadrupeds and between quadrupeds and humans, it remains unclear whether common model animals can simulate capsulolabral pathology that occurs in humans. While the human labrum and capsule have been extensively characterized [3, 9, 10, 17, 26, 37, 38], similar analyses in animal shoulders are scarce and incomplete [1, 5, 27, 34, 35]. Before novel strategies for improved capsulolabral healing can be translated from animal models to human patients, an understanding of the similarities and differences in the native glenolabral structure and function across species is needed. Therefore, the purpose of this study was to characterize the anatomic and histological properties of the shoulder labrum in species commonly employed as animal models, including rat, rabbit, dog, pig, and goat. Given the demonstrated similarities in size and structural morphology in other joints [11, 31], it was hypothesized that the goat glenoid with surrounding capsulolabral complex would most closely resemble that of humans in terms of dimensions and structure, as observed grossly and histologically.

## Methods

Approval was obtained from the Committee for Oversight of Research and Clinical Training Involving Decedents (*BLINDED*) from *BLINDED*. Cadaveric glenohumeral joints were freshly harvested from skeletally mature animals from five species commonly utilized as animal models for shoulder injury and repair, including rats (*n* = 8), New Zealand white rabbits (*n* = 13), Mongrel dogs (*n* = 9), Spanish goats (*n* = 10), Yorkshire pigs (*n* = 10), and humans (*n* = 9). All soft tissue surrounding the joint was dissected without violating the joint capsule. Thereafter, the capsule was sharply dissected free from its humeral insertions.

**Fig. 1:**
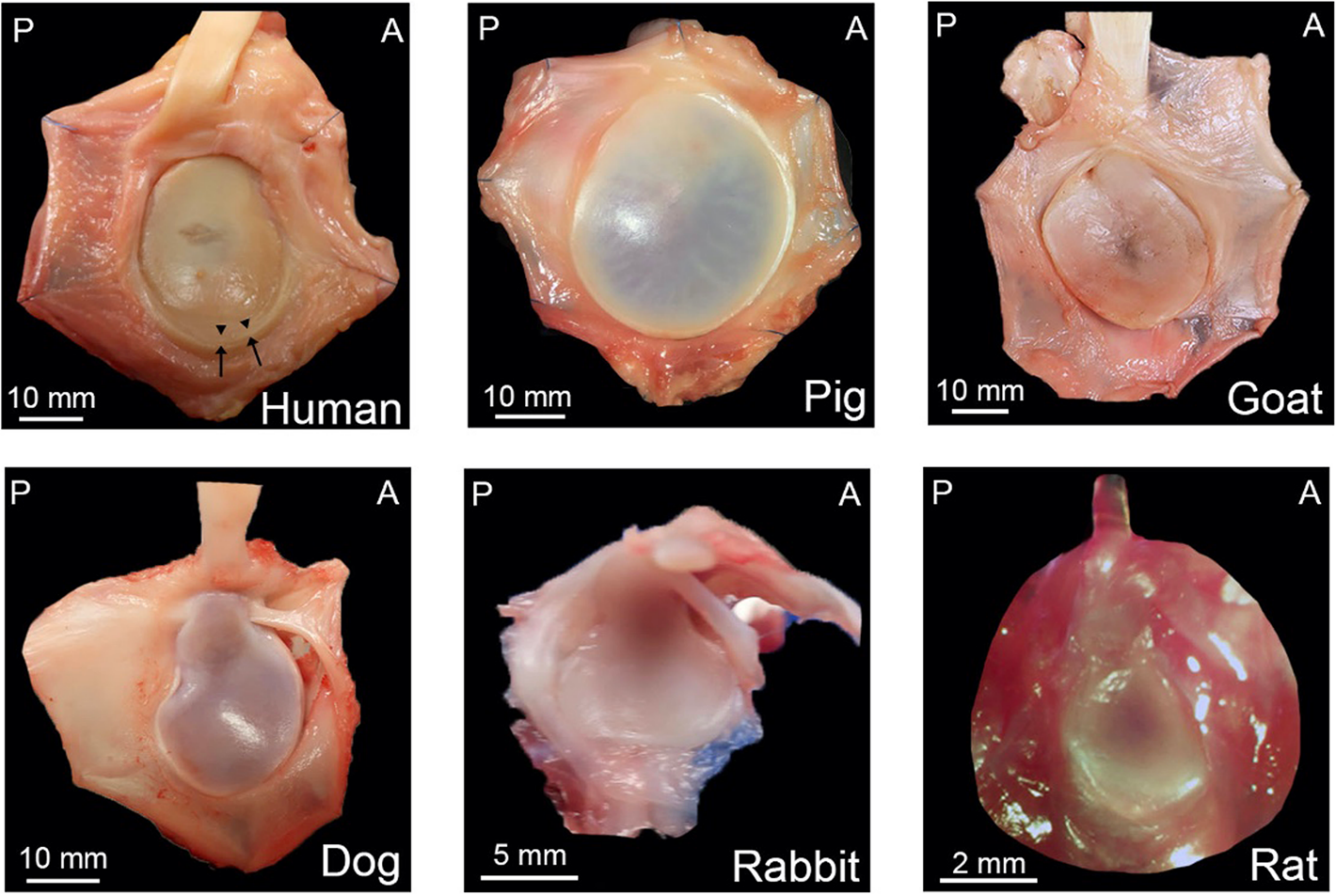
Gross anatomy of the glenoid with attached capsulolabral complex. Anterior (A) on right, posterior (P) on left, in each panel. Superior is top of panel, with 12 o’clock position indicated by the inserting long head of biceps. Glenolabral and capsulolabral junctions in the human shoulder indicated by arrowheads and arrows, respectively; absent in other animals

**Fig. 2:**
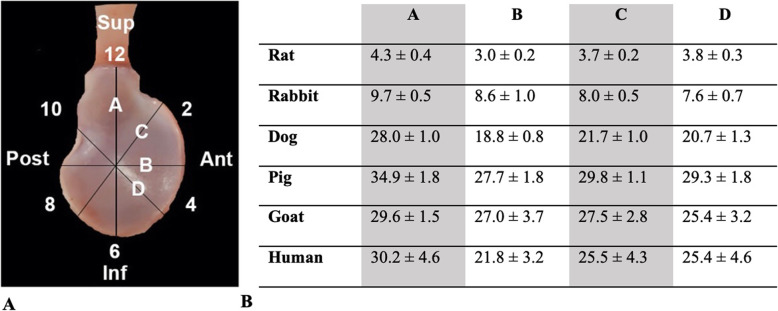
**A** Glenoid clockface positions with **B** linear dimensions (in mm)

**Fig. 3:**
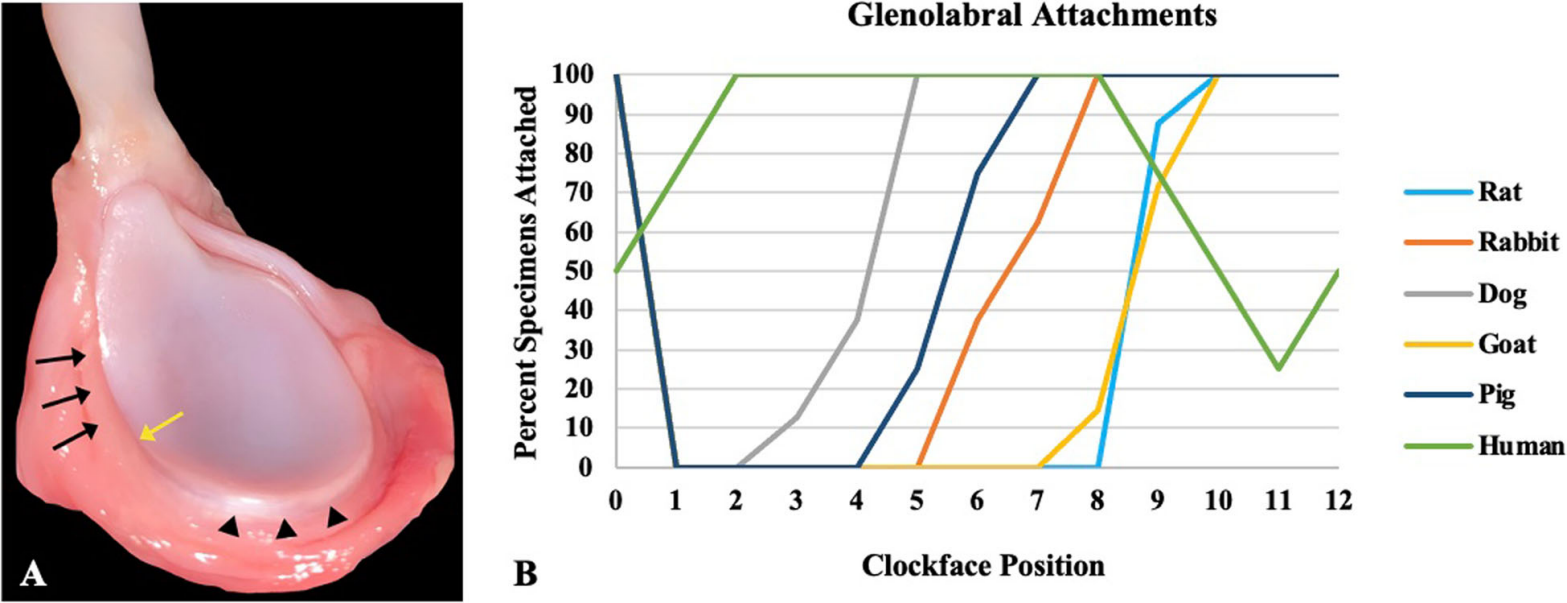
**A** Delineation of continuous and recessed capsulolabral complex from the articular glenoid surface. Black arrows indicate continuous complex; arrowheads indicate recessed complex; yellow arrow indicates transition point between continuous and recessed complex. **B** Percent of specimens (by species) in which the capsulolabral complex was continuous with the glenoid articular surface

Photographs were taken of the glenoid *en face* with its surrounding capsulolabral complex, defined as the soft tissue inserting immediately into the glenoid (Fig. 1). Linear dimensions of the articular surface of the glenoid were determined using digital calipers along four lines dissecting the glenoid (Fig. 2A). According to a clock face orientation (Fig. 2A), it was determined where the capsulolabral complex was continuous with, or recessed from (i.e., soft tissue inserted towards the glenoid neck), the articular glenoid surface (Fig. 3A). The insertion of the long head of the biceps tendon into the supraglenoid tubercle designated the 12 o’clock position, as it was present in all specimens of all species. The glenoid was then divided into six equal segments radiating out from the glenoid center toward 12, 2, 4, 6, 8, and 10 o’clock positions in order to prepare samples for histology.

Samples were fixed in 10% formalin. Each glenoid was then decalcified in formic acid with ethylenediaminetetraacetic acid (Formical2000, Thermo Fisher, Pittsburgh, PA, USA), serially dehydrated in a graded ethanol series, and embedded in paraffin. Each glenoid segment was sectioned in a plane orthogonal to the glenoid surface and capturing the interface between the glenoid articular surface and labrum/capsule. The segments were cut into 7 μm slices by a Leica RM255 microtome (Leica Biosystems, Buffalo Grove, IL, USA). Histological sections were stained with either Mallory Trichrome (Electron Microscopy Sciences, Hatfield, PA, USA) or Safranin O/Fast Green (Electron Microscopy Sciences) according to standard histological protocols and photographed using an Olympus SZX16 stereoscope mounted with a SPOTFlex FX1520 camera (Olympus, Center Valley, PA, USA) [32, 33]. Insertion of the capsulolabral tissue onto the glenoid was qualitatively assessed and compared with gross morphology.

Unless otherwise indicated, data are displayed as mean ± standard deviation. Statistical analyses were performed using SPSS 27.0 software (IBM, Armonk, NY, USA). A two-way analysis of variance with post-hoc Tukey correction was performed to evaluate for differences among glenoid dimensions between and within species. Significance was set at *P* < .05.

## Results

On gross inspection, the posterior capsule was qualitatively thicker than the anterior for all species (Fig. 1). When comparing dimensions within species, the superior-inferior (line A) and anterior-posterior (line B) dimensions demonstrated that the glenoid was taller than wide (A > B) in all animals (*p* < .005) except goats (*p* = .20). The anterosuperior-posteroinferior (line C) and anteroinferior-posterosuperior (line D) dimensions were equivalent (C = D) in all animals (Fig. 2). When comparing dimensions across species, line A was a similar length in dogs and goats (*p* = .58), dogs and humans (*p* = .27), and dogs and goats (*p* = .99). Line B was a similar length among dogs and humans (*p* = .07) and goats and pigs (*p* = .97). Line C was similar between pigs and goats (*p* = .15) as well as goats and humans (*p* = .35). Line D was similar between goats and humans (*p* = 1.0). All other combinations were significantly different across species (*p* < .05).

**Fig. 4:**
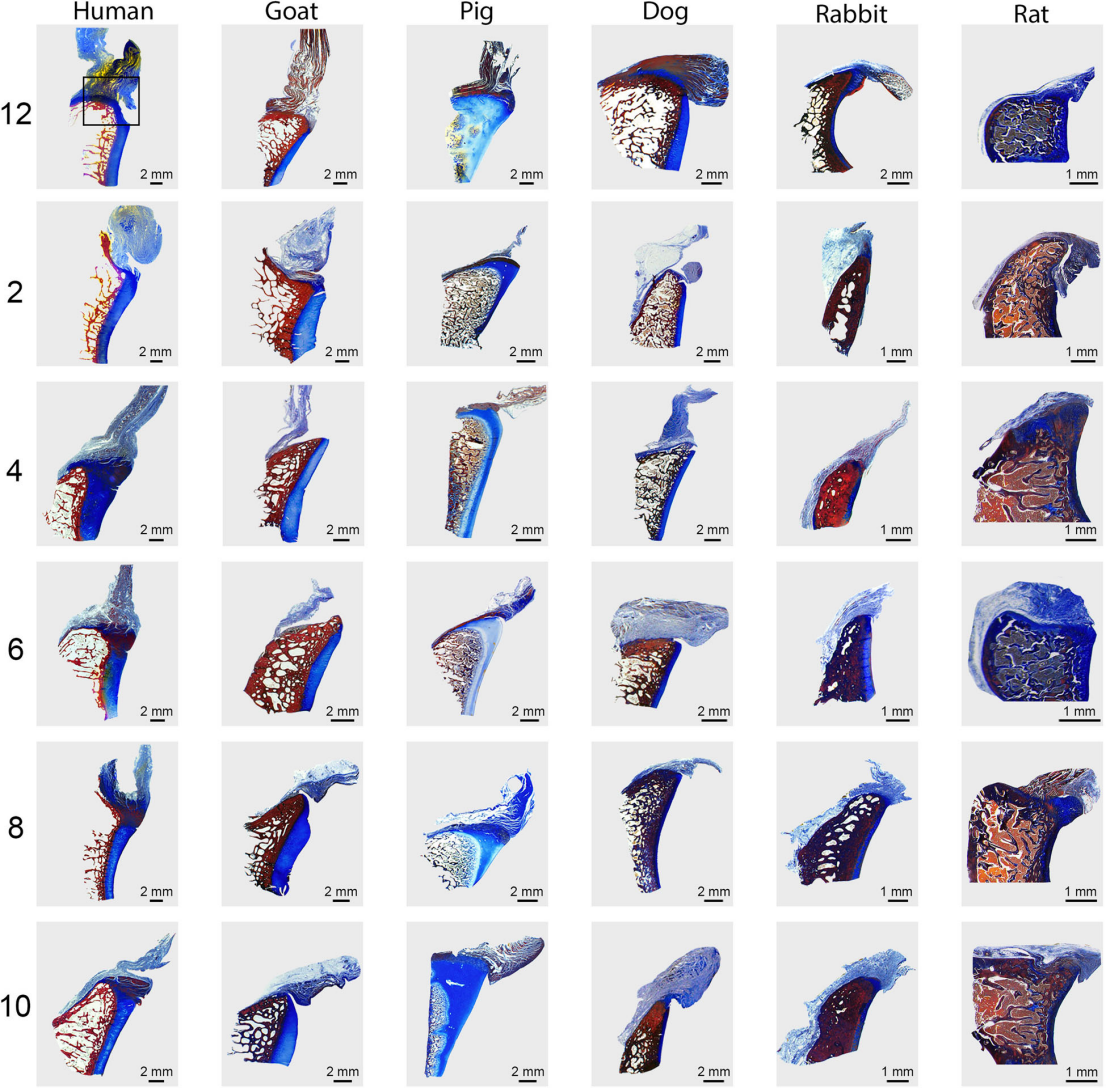
Lower magnification Mallory trichrome-stained sections of the glenocapsular junction at each clockface position across species. Box on human 12 o’clock image (top left) indicates higher magnification region of interest shown in Fig. 5

**Fig. 5:**
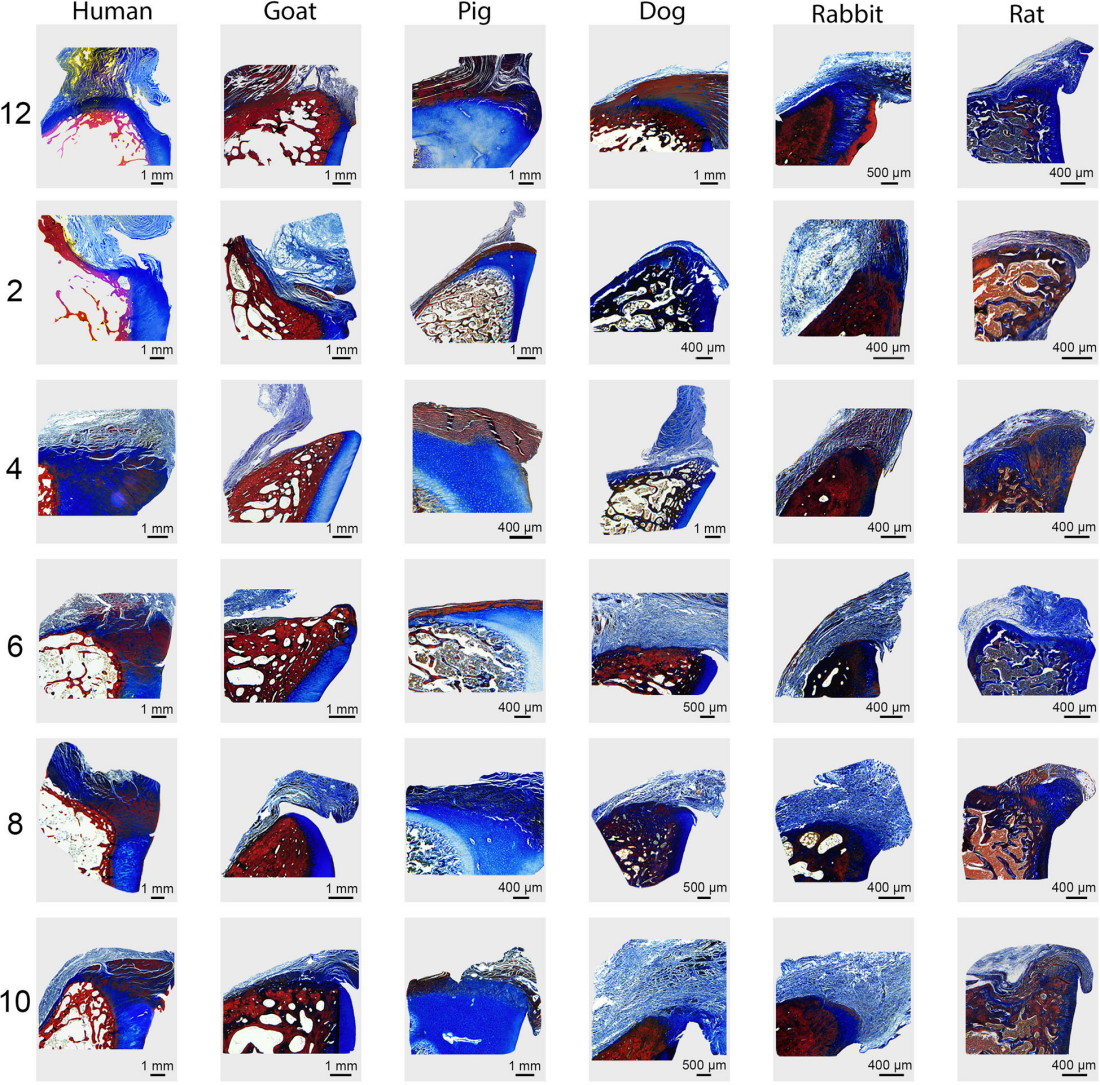
Higher magnification Mallory trichrome-stained sections of the glenocapsular junction at each clockface position across species

The capsulolabral complex was continuous with the glenoid surface from ~ 2 to 8 o’clock in humans, 6 to 12 o’clock in pigs, 9 to 12 o’clock in goats, 5 to 12 o’clock in the dogs, 7 to 12 o’clock in rabbits, and 9 to 12 o’clock in the rats, as shown on both gross anatomy and histology (Figs. 1 and 3B). In contrast to humans, no other species demonstrated an organized fibrocartilaginous labrum either grossly or histologically. Low and high magnification photographs of histological staining with Mallory trichrome or Safranin O/Fast green are shown in Figs. 4 and 5, and Supplemental Figures 1 and 2, respectively.

## Discussion

**Fig. 6:**
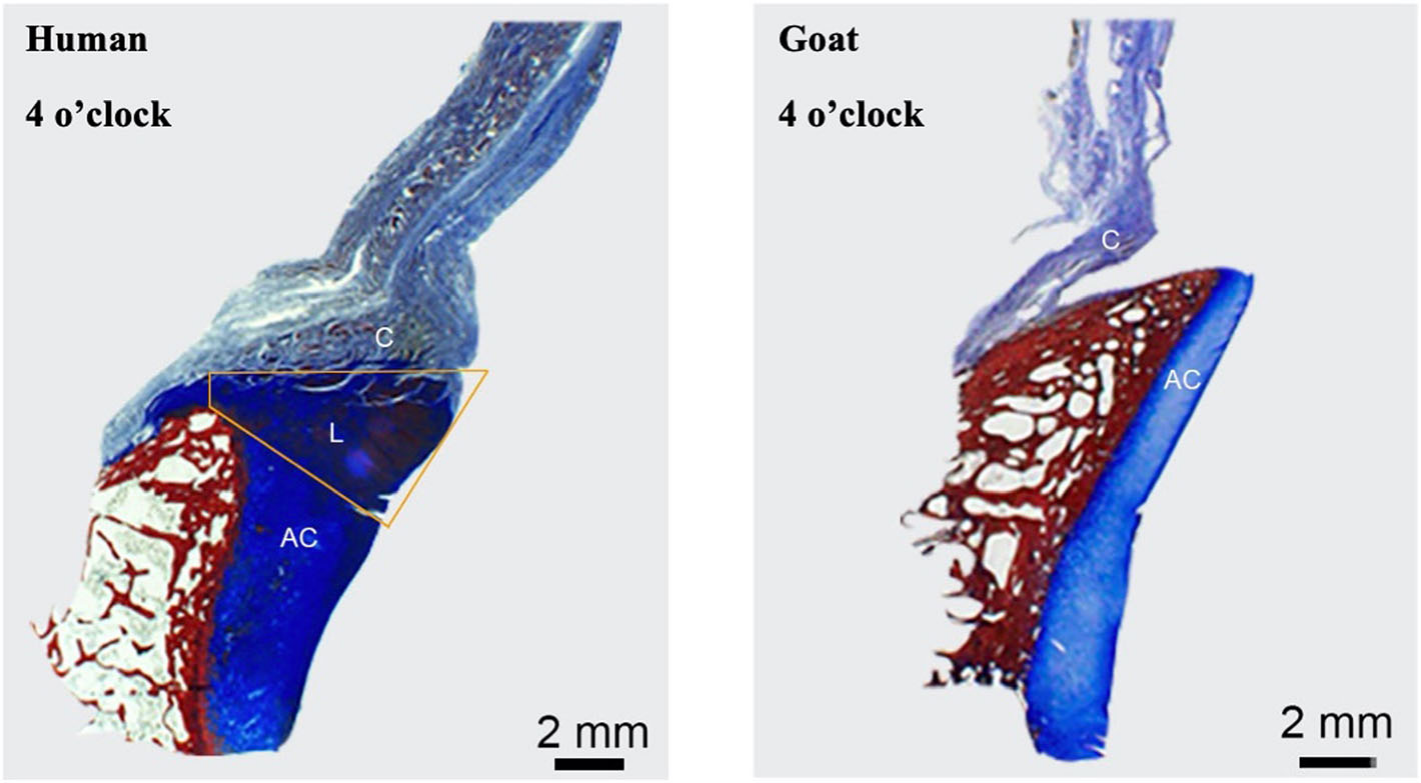
Attachment site comparison between human (left) and goat (right) at 4 o’clock position, exemplifying the presence and absence of a discrete labral structure in humans compared to other animal species, respectively. Articular cartilage (AC), labrum (L), and capsule (C)

The most important finding of this study was that none of the included animal species possessed a distinct fibrocartilaginous glenoid labrum discernible by either gross or histological evaluation, as compared to the human glenoid labrum (Fig. 6). The hypothesis that goats would be most similar to humans was partially supported, as the dimensions of the goat glenoid were most similar to that of humans, yet goats did not possess a true labrum and had different attachment sites for the capsulolabral complex.

The human labrum and capsule have been extensively studied [3, 9, 10, 17, 26, 37, 38], but similar analyses of other species are scarce and inconsistent [1, 5, 21, 27, 34]. While non-human primates ostensibly possess shoulder anatomy closest to humans given our relatively recent evolutionary divergence, the use of non-human primates is expensive and increasingly tempered by ethical considerations. As with most animal models, studies of the structure and function of the non-human primate shoulder have focused extensively on the rotator cuff rather than the capsulolabral complex [28]. Additionally, an investigation of the effect of anterior shoulder dislocation on cadaveric simian shoulders only characterized the capsule and not the labrum [8]. Similarly, in a sheep model of capsular plication, effects on the labrum were not described [19].

Of the large animal models, the capsulolabral complex of the dog has been best characterized. In a study similar to the present, the labrum and capsular ligaments of the dog shoulder were characterized grossly and histologically according to clock face positions [35]. However, a direct comparison to human anatomy was only performed for gross morphology using formalin-fixed dog specimens. While a discrete labrum in dogs was described, fixation can often increase the apparent thickness of a multiple-layered capsule so as to give the appearance of a distinct structure [14]. In this prior study, only dog specimens were prepared for histology and there was inconsistent demonstration of a fibrocartilaginous labrum at each clockface position. These findings, with the aforementioned limitations, contrast the findings of the present study which did not find a discrete fibrocartilaginous labrum at any position, in any specimen, of the dog glenoid.

Approaching greater clinical relevance, albeit with a smaller animal, an anteroinferior labral injury (i.e., Bankart lesion) was simulated in a rabbit model by sharp incision of the capsulolabral complex at the glenoid edge [1]. Biomechanical and histological analyses were then performed at sequential timepoints to assess capsulolabral healing. While neotissue formation at the iatrogenic defect was demonstrated over time, a distinct fibrocartilaginous labrum was not apparent on the histological images. In related studies of a simulated anteroinferior dislocation in a rat model, the capsulolabral complex was incised at the glenoid junction and the humeral head was manually dislocated [25, 27]. As with the rabbit model above [1], histology demonstrated neotissue formation at the capsule-glenoid junction, but no distinct fibrocartilaginous labrum was clearly discernible. As suggested above, the past literature has largely assumed the presence of a distinct fibrocartilaginous labrum in animal models of labral injury and healing, yet review of the results does not conclusively support this position. In contrast, this study thoroughly evaluated the putative glenoid-labral interface of five model animals using morphological and histological analyses and found no distinct fibrocartilaginous labrum as clearly demonstrated in human shoulders. It is also notable that the capsulolabral complex was continuous with the glenoid surface anteriorly in humans, but not in any animal species. This unique feature of human shoulders may in part be due to differences in locomotion (i.e., bipedal vs. quadrupedal) and the increased propensity for anterior dislocation of the human shoulder, while acknowledging that the incidence and injury patterns of shoulder instability in animals is largely unreported.

This study is not without limitations. Similar to the existing literature on the topic, the results presented herein are largely qualitative and therefore subjective. While subjectivity is often inherent in the assessment of gross morphology and histology, the inclusion of a human specimens provided a positive control for defining the presence or absence of a fibrocartilaginous labrum in model animals. Nevertheless, inclusion of additional stains or immunohistochemistry may have further supported distinct structural and biochemical differences in the capsulolabral complex of humans and the included animal species. The five species of animals investigated herein are among the most commonly used for models of shoulder pathology, but similar characterization of additional species, including non-human primates, would be useful. The focus of this study was the characterization of the labrum and glenoid-labrum interface; while inspection of the capsule for glenohumeral ligaments was performed during gross dissection, these data were not formally included herein. Future characterization of the glenohumeral ligaments and other static stabilizers will be important for ongoing development of novel animal models, as these structures likely play a vital role in glenohumeral stability, especially in the absence of a discrete labrum, and in keeping with the conceptualization of the static stabilizers as a periarticular fiber system of the shoulder [15]. Furthermore, as we continue to learn about the synergistic relationships of passive and dynamic stabilizers of the human shoulder, parallel studies in animal models may illuminate how animals, absent a labrum, can have largely stable shoulders.

## Conclusion

The species examined in the present study did not possess a labrum by gross or histological evaluation. While models using these animals may be acceptable for examining other shoulder pathologies, they are not adequate to evaluate labral pathology.

## Supplementary Information

**Additional file 1: Supplemental Figure 1.** Lower magnification safranin-O-stained sections of the glenocapsular junction at each clockface position across species. Box on human 12 o’clock image (top left) indicates higher magnification region of interest show in Supplemental Figure 2.

**Additional file 2: Supplemental Figure 2.** Higher magnification Safranin-O-stained sections of the glenocapsular junction at each clockface position across species.

## Data Availability

The datasets used and/or analysed during the current study are available from the corresponding author on reasonable request.
