# Supplementary figures and images for: Common animal models lack a distinct glenoid labrum: a comparative anatomy study
Source: J Exp Orthop. 2021 Aug 16;8:63. doi: 10.1186/s40634-021-00383-6 (PMC8368311; doi:10.1186/s40634-021-00383-6)

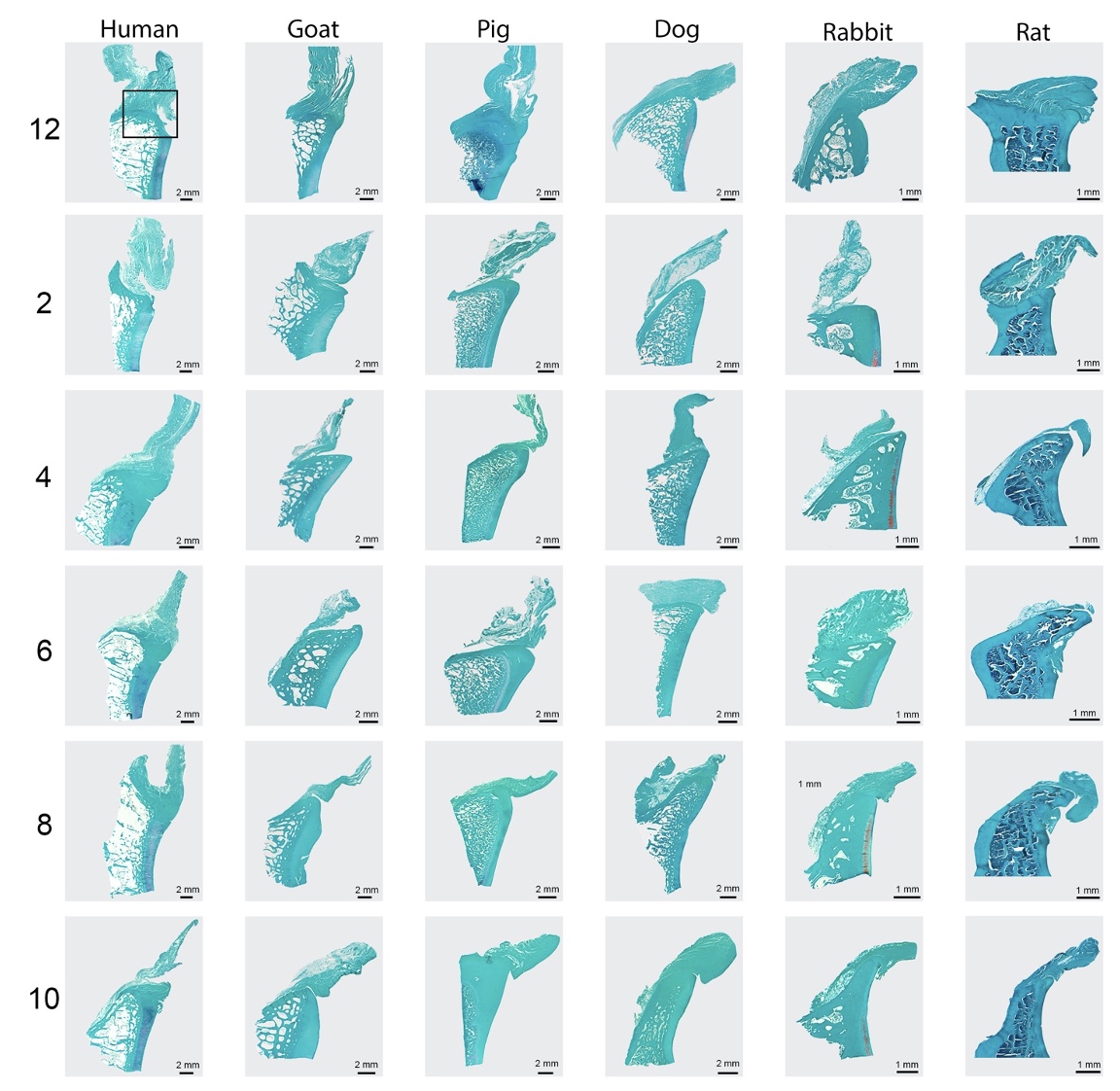

Supplement: Supplementary file 1 — Additional file 1: Supplemental Figure 1. Lower magnification safranin-O-stained sections of the glenocapsular junction at each clockface position across species. Box on human 12 o’clock image (top left) indicates higher magnification region of interest show in Supplemental Figure 2. [file 40634_2021_383_MOESM1_ESM.jpg]

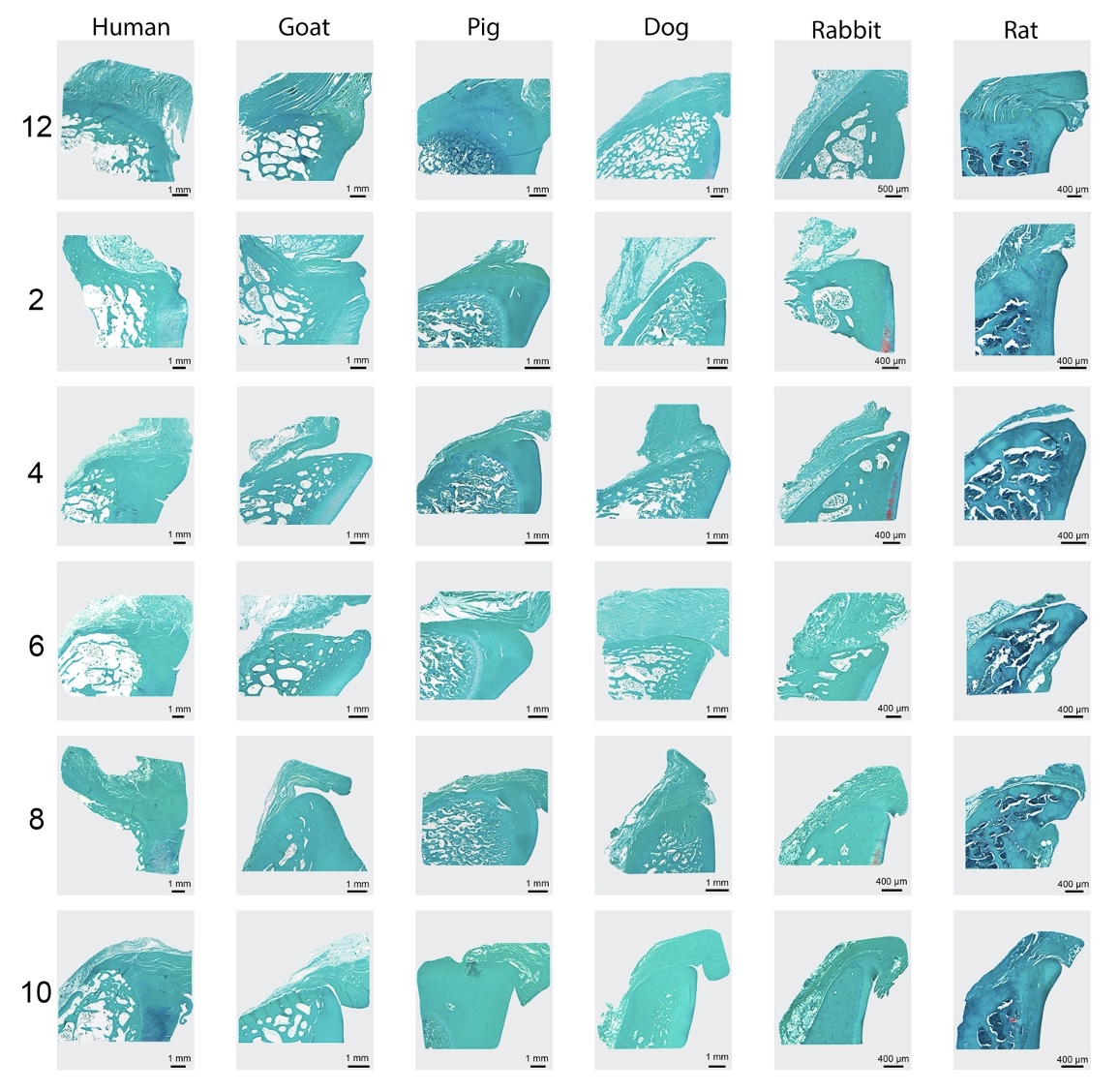

Supplement: Supplementary file 2 — Additional file 2: Supplemental Figure 2. Higher magnification Safranin-O-stained sections of the glenocapsular junction at each clockface position across species. [file 40634_2021_383_MOESM2_ESM.jpg]
